# Native and non-native winter foraging resources do not explain *Pteropus alecto* winter roost occupancy in Queensland, Australia

**DOI:** 10.3389/fevo.2024.1483865

**Published:** 2024-10-18

**Authors:** Kelsee Baranowski, Nita Bharti

**Affiliations:** Department of Biology, Center for Infectious Disease Dynamics, The Pennsylvania State University, University Park, PA, United States

**Keywords:** *Pteropus*, diet, seasonal habitat, resource reliability, Hendra virus

## Abstract

Anthropogenic land use change concurrent with introductions of non-native species alters the abundance and distribution of foraging resources for wildlife. This is particularly concerning when resource bottlenecks for wildlife are linked to spillover of infectious diseases to humans. Hendra virus is a bat-borne pathogen in eastern Australia. Spillovers align with winter food shortages for flying foxes and flying foxes foraging in agriculture or peri-urban lands, as opposed to native forests. It is believed the increased abundance and spatiotemporal reliability of non-native species planted in anthropogenically modified areas compared to native, ephemeral diet species may be a key draw for flying foxes into urban and peri-urban areas. We investigate the explanatory power of environmental factors on the winter roost occupancy of the reservoir for Hendra virus, the black flying fox *Pteropus alecto*, from 2007–2020 in Queensland, Australia. We measured the extent, spatial aggregation, and annual reliability of typical (i.e. native) and atypical (i.e. non-native) winter habitat species in 20km foraging areas around roosts surveyed by the National Flying Fox Monitoring Program. We find that neither the extent nor the spatial distribution of winter habitats explained black flying fox winter roost presence. Although the establishment of roosts was associated with high reliability for typical winter diet species, the reliability of frequently listed winter diet species surrounding surveyed roosts was not different between roosts that were occupied versus unoccupied in the winter. Significant interactions between lagged weather conditions and winter habitats identified by the best model did not reflect observable differences in patterns of occupancy upon scrutiny. Static measures of winter habitat and weather conditions poorly explained the winter roost occupancy of black flying foxes. Understanding the drivers of flying fox movement and presence requires further investigation before they can be thoughtfully integrated into Hendra spillover prevention efforts and flying fox management.

## 1 Introduction

Environmental conditions and resource availability are key determinants of the distribution of wildlife populations across a landscape (Orians and Wittenberger, 1991; López-López et al., 2014; Baruch-Mordo et al., 2014). Individuals are likely to be found in and move to locations where the probability of encountering resources is high (Stephens and Charnov, 1982; Jones et al., 2014). Resources can vary across space or through time, and the spatial aggregation, availability, and predictability of high-quality resources can all influence wildlife foraging movements (Pyke, 1984; Saracco et al., 2004; Jonzén et al., 2011; Riotte-Lambert and Matthiopoulos, 2020). Optimal foraging strategies suggest individuals will try to maximize nutritional benefits while limiting energetic costs (Stephens and Charnov, 1982; Pyke, 1984) and reducing the risk of predation (Lima and Dill, 1990). Anthropogenic land use change creates novel landscapes for animals to forage in, and ultimately alters the availability and distribution of foraging habitats for wildlife (Corlett et al., 1992; Foley et al., 2005; Bradshaw, 2012; Chase et al., 2020).

Across diverse taxa, animal populations are changing their foraging behavior in response to anthropogenically modified landscapes. Migratory seabirds forage in landfills (Corman et al., 2016) and fishery discards (Patrick et al., 2015). Black bears (*Ursus americanus*) forage in urban areas, regardless of native food resource availability (Merkle et al., 2013). Similarly, Australian flying foxes (*Pteropus* sp.) are urbanizing and changing their foraging behaviors, perhaps in response to profound environmental changes (Tait et al., 2014; Meade et al., 2021; Yabsley et al., 2021). This is particularly concerning, considering the beneficial role they play as pollinators (Eby, 2016) in the heavily cleared native eucalypt forests (Bradshaw, 2012) and the zoonotic disease risks they present as reservoir hosts for viruses, such as bat lyssaviruses and Hendra virus (Eby et al., 1999; Plowright et al., 2011; Mahalingam et al., 2012; Eby, 2016; Peel et al., 2022).

Australian flying foxes are nomadic, nocturnal nectivores and frugivores that roost in canopy vegetation during the day (Nelson, 1964; Marshall, 1985; Eby, 1991). They have typically foraged on small fruits such as figs and nectar from members of the Proteaceae and Myrtaceae families, notably *Eucalyptus, Melaleuca, Banksia*, and *Corymbia* species (Ratcliffe, 1931; Nelson, 1964; Marshall, 1985; Palmer, 1997; Markus and Hall, 2004; Eby and Law, 2008). Flying foxes historically made long-distance movements (>300 kilometers (km)) across their range to follow the seasonal flowering of their diet resources (Eby, 1991; Roberts et al., 2012), forming aggregations larger than 100,000 individuals when nectar was in great abundance (Ratcliffe, 1931). Recently, these large aggregations have become less common (Eby et al., 2023), and flying foxes have been observed roosting in smaller groups with roosts becoming more numerous across eastern Australia (Baranowski and Bharti, 2023), particularly after winters when typical diet resources were scarce (Eby et al., 2023).

Studies of flying fox diets reveal insights into their recent changes in foraging ecology. Meade et al., 2021 found grey-headed flying foxes (*Pteropus poliocephalus*) in urban areas primarily forage on a mixture of native and anthropogenic resources, including exotic species. Similarly, the availability of human-planted urban resources can attract flying foxes to areas beyond their historic range, such as the Melbourne and Adelaide Botanic Gardens (Van Der Ree et al., 2006; Yabsley et al., 2021). Studies that tracked black flying foxes (*P. alecto*) with radio telemetry in the urban center of Brisbane showed no individuals fed on any eucalypt species in winter (Markus and Hall, 2004). Rather, animals foraged on native and invasive fig species (*Ficus* sp.), and invasive Cocos palm (*Syagrus romanzoffiana)*. Field et al. similarly found black flying foxes primarily fed in fragmented lands on weedy species, such as climbing asparagus vine (*Asparagus africanus*) and wild tobacco (*Solanum mauritianum*), as opposed to a typical blossom diet when foraging from a roost with little forest nearby (Field et al., 2016).

It is hypothesized this shift in resource consumption is driven by the increased spatiotemporal availability and reliability of these atypical diet resources (Welbergen et al., 2020; Meade et al., 2021; Yabsley et al., 2022), over typical *Eucalyptus* diet resources, which may flower only once over 1–5 years, depending on the species (Law et al., 2000). Longitudinal studies of flying foxes from 2012–2017 found their foraging movements were more quasi-random, and individuals could be drawn to urban or forested areas that are temporarily productive (Welbergen et al., 2020). These recent observations of flying fox winter foraging ecology are particularly concerning as they could indicate increased contact rates between human systems and black flying foxes, the main reservoir responsible for spillovers of Hendra virus.

Hendra virus is a zoonotic paramyxovirus that spills over from *Pteropus* bats to horses (*Equus caballus)* and subsequently to humans and other domestic animals (Murray et al., 1995; Plowright et al., 2011; Mahalingam et al., 2012). Nearly all known spillover events through 2023 (64/66) have occurred within the range of the black flying fox along the eastern coast of Queensland and northeastern New South Wales, Australia (Queensland Government Business Queensland, 2021). Two-thirds of all documented Hendra virus spillovers have occurred within the Austral winter months of June, July, and August, which coincides with a scarcity of typical native diet resources (Eby and Law, 2008). Studies on Hendra virus have linked winter food shortages (Becker et al., 2022), poor body condition (Edson et al., 2019), and flying foxes overwintering at new roosts containing less native winter habitat than historic overwintering roosts, (Becker et al., 2022) with greater detection of viral RNA in flying foxes (Edson et al., 2019) or their excreta (Becker et al., 2022). However, given the changes in flying fox movement and foraging ecology, it’s unclear how winter diet resources in roost foraging areas influence black flying fox winter distribution and roost occupancy.

We assess the relationships between typical and atypical winter foraging habitats, monthly weather conditions, and the winter roost occupancy of black flying foxes in Queensland from 2007 to 2020. We focus on these years due to the notable increase in the frequency of Hendra virus spillovers (Queensland Government Business Queensland, 2021). Given the findings from prior foraging studies on flying foxes, we hypothesized that black flying fox winter roost occupancy would be positively correlated with roosts containing abundant and annually reliable typical and atypical winter habitats. We also hypothesized that roosts with dispersed winter diet resources would be occupied more frequently than roosts with aggregated resources in their foraging areas, as black flying foxes have been noted to preferentially forage in fragmented forests (Field et al., 2016). We find that the location of roosts was associated with high reliability for typical winter diet species but the presence of black flying foxes at roosts in winter was not driven by the abundance, spatial clustering, or reliability of native or non-native winter habitats. Modeling the interactions of temporally lagged weather conditions on winter habitats identified no consistent environmental variables that explained black winter roost occupancy in Queensland.

## 2 Methods

**FIGURE 1:**
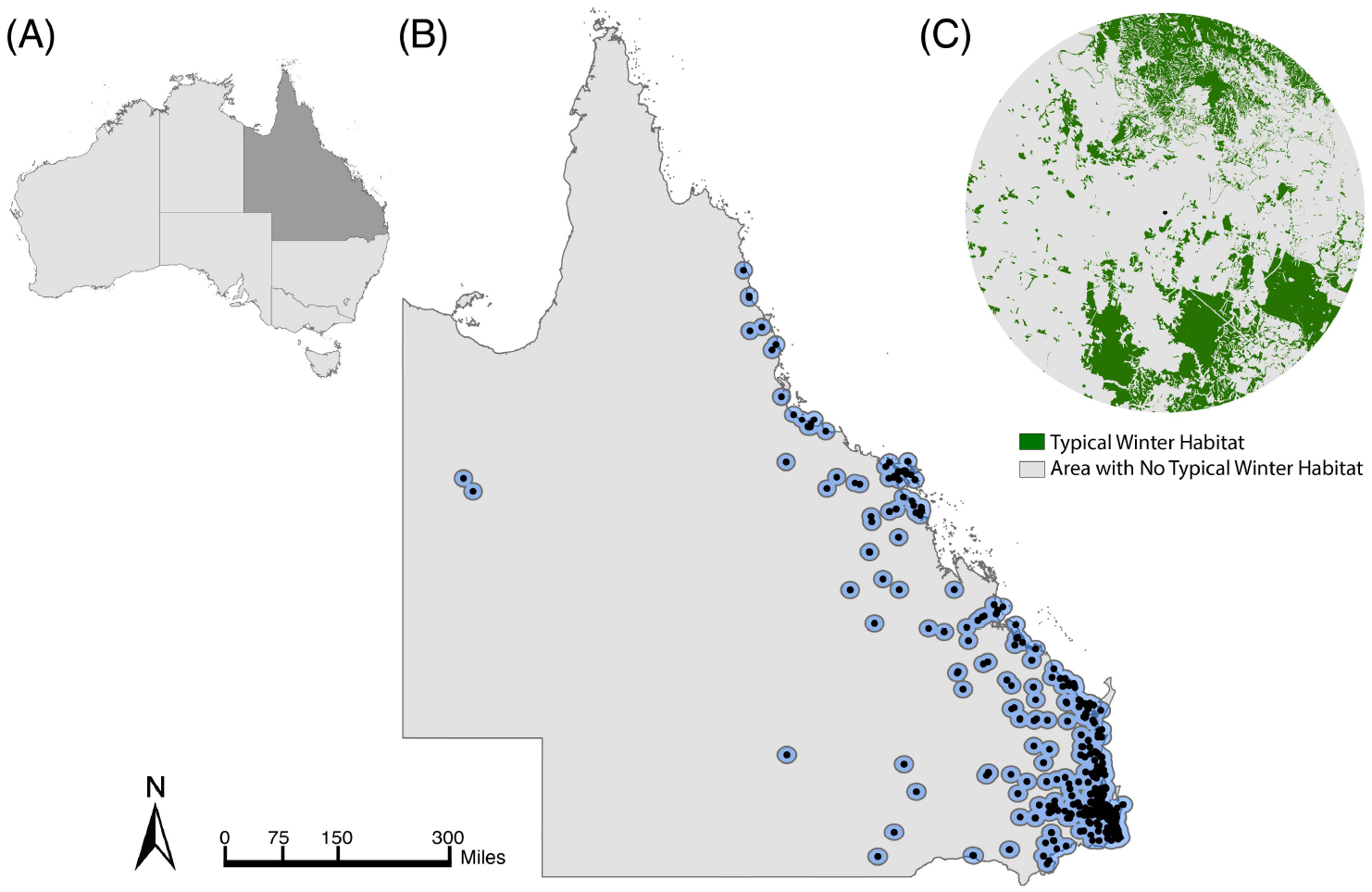
Study area of Queensland, Australia. (**A**) The country of Australia with the state of Queensland shaded in dark grey. (**B**) Map of Queensland with black points identifying flying fox roosts surveyed in winter between 2007 and 2020 and blue circles denoting the boundaries of each roost’s 20 km radius foraging buffer. (**C**) Example roost’s 20 km foraging area, with areas containing typical winter diet species in green and areas without winter diet species in grey.

We used flying fox roost surveys in Queensland from the National Flying Fox Monitoring Program (NFFMP) (Australian Government of Agriculture, Water and the E, 2021) to identify monitored roosts that were used by black flying foxes from 2007–2020 (Supplementary Figure S1). These surveys, completed by government employees and volunteers, produce serial animal counts for nationally known or established roosts. They focus on roosts used by grey-headed and spectacled flying foxes (*P. conspicillatus*) because these species are listed as ‘Vulnerable’ and ‘Endangered,’ respectively (Westcott et al., 2015) but counts of all *Pteropus* species present are recorded. The species-specific roost selection may bias the data to overlook roosts occupied solely by black flying foxes. However, black flying foxes and grey-headed flying foxes often co-roost (Welbergen et al., 2020). Over 97% (266/274) of the roosts occupied by grey-headed flying foxes in Queensland were also occupied by black flying foxes in at least one roost survey from 2007–2020. We present results for the estimated average foraging area, a 20-kilometer radius buffer, which is based on foraging flights measured with radio and satellite telemetry (Roberts et al., 2012), equaling 1256.64 km^2^, around each flying fox roost (n=455) surveyed by the NFFMP (Figure 1B). See Supplementary Figures S2, S3 for analysis of additional buffer sizes.

### 2.1 Flying fox diet

Data on black flying fox diet species were collected from numerous studies on black flying fox diet and foraging studies in Queensland and northern New South Wales (Ratcliffe, 1931; Markus and Hall, 2004; Eby and Law, 2008; Field et al., 2016; Eby et al., 2019; Griffith, 2020; Bell et al., 2021; Bradford et al., 2022). Diet species were categorized as *typical* and *atypical* species, based on the genus and the relative frequency with which black flying foxes were observed foraging on each species in the earliest reports of black flying fox foraging ecology by Ratcliffe in 1931 (Ratcliffe, 1931). He stated, “The principal food of flying foxes (all species) in Australia is undoubtedly blossom” and specified flying foxes predominantly fed on the nectar and blossom of native species in the genera *Eucalyptus, Banksia, Melaleuca, Castanospernum*, and the fruit of *Ficus* species. He also noted flying foxes foraged on rainforest and commercial fruits when native resources were depleted. We define *typical* winter diet species as those observed in Ratcliffe, 1931 and other flowering tree species belonging to the genera *Eucalyptus, Banksia, Melaleuca, Castanospernum*, and *Ficus*. We define *atypical* winter diet species and any blossom or fruit diet species outside these genera on which black flying foxes or grey-headed flying foxes were observed foraging in winter (e.g. *Syagrus*, *Diospyros, Solanum*). See Supplementary Table S1 for a complete list of winter diet species.

We used the Queensland Herbarium’s Vegetation Management Regional Ecosystem (VMRE) maps (Queensland Government, 2014; Queensland Herbarium, 2019) to locate native (typical and atypical) winter diet species for black flying foxes biennially from 2007–2019. Regional ecosystems are vegetation communities within a bioregion that are consistently associated with a particular combination of geology, landform, and soil (Neldner et al., 2019). The VMRE maps integrate data from field surveys, aerial photography, and satellite imagery, as well as geology, soil mapping, historical surveys, etc. (Neldner et al., 2019). These products map the remaining remnant, or primary native, vegetation in Queensland every two years from 1997 to 2019. We associated these maps with the Regional Ecosystem Description Database (Queensland Herbarium, 2019) to gain highly detailed information about native black flying fox seasonal diet species dynamics over time. However, these data do not map the abundance or distribution of non-native vegetation.

We used the Global Biodiversity Information Facility (GBIF) database to identify locations where typical and atypical black flying fox diet species have been identified by sources other than the Queensland Herbarium, particularly non-native species. GBIF incorporates records of species locations from national datasets, like the Australia Living Atlas, as well as crowd-sourced data, such as iNaturalist. These records are point locations of tree species, rather than polygons of vegetation communities as in the VMRE. Although these data may be spatially biased towards areas readily accessible by people, they provide important supporting information about the location of atypical winter diet species not considered in the VMRE map, given their non-native vegetation status. We removed observations of trees classified as ‘Preserved Specimen’ or ‘Machine Identification’ and retained records of live trees that were identified by a person. We also removed records with fewer than two decimal degrees of spatial resolution in the coordinate fields due to locational uncertainty and eliminated repeated observations. We removed observations outside a 100km radius of any flying fox roosts and created a derived dataset of these records (Derived dataset GBIF.org, 2024).

We buffered each winter diet tree record in GBIF by 8 meters to transform a single point to a representative canopy polygon based on a median projected canopy area from six *Eucalyptus* species ranging in size from 132m^2^ to 230m^2^ (Verma et al., 2014). We selected tree records observed before 2000 and spatially overlaid these with the National Forest and Sparse Woody Vegetation (Department of Environment and Science, 2020) map from 2000. We removed observations identified before 2000 that were not in a pixel considered woody vegetation in this dataset to limit the inclusion of trees that were likely cleared before our study period.

We merged the vegetation from the regional ecosystems and the GBIF data to represent the breadth of typical and atypical winter diet species around flying fox roosts in Queensland. We recorded the frequency of winter diet species listed in vegetation community descriptions and only considered the top six most prevalent species. We further recorded the annual reliability, or the annual likelihood of flowering or fruiting, of these diet species when available (House, 1992; Westcott et al., 2005; Eby and Law, 2008; Schmelitschek et al., 2009; Business Queensland, 2021). We intersected the merged biennial vegetation maps with the 20 km buffers around roosts surveyed by the NFFMP (Figure 1B) to quantify the extent of patches that contain typical or atypical winter diet species for black flying foxes (Figure 1C). We generated a hull enclosing all roosts sampled by the NFFMP in eastern Queensland. We used the Generate Random Point tool in ArcGIS Pro to generate 500 random points within the hull that fell outside of the roost 20km foraging buffer, with at least 1km of distance between generated points, and randomly selected 125 (25%) of those for further analysis.

### 2.2 Spatial arrangement of winter habitats

We measured the Global Moran’s I value (Moran, 1950) for winter habitats in roost foraging areas. Moran’s I values range from −1 to 1 and can indicate spatial dispersion or clustering. Significant correlations between the spatial aggregation of fruit resources and frugivory have been observed with frugivorous birds (Saracco et al., 2004). The spatial concentration of anthropogenic resources is also thought to be a key contributing factor in black flying fox urban foraging behavior (Meade et al., 2021). We used Moran’s I to determine whether the spatial aggregation of winter habitats influenced black flying fox winter roost occupancy. We classified each roost’s 20 km foraging buffer into areas containing winter habitat (typical or atypical) and areas not containing winter habitat. We measured the Euclidean distance between the centroids of all polygons containing winter habitat. We defined neighbors as type Queen and used binary coding for the neighbor weight matrix. We used the moran.mc function to run 500 Monte-Carlo simulations for each roost’s Moran’s I value from the *spdep* package (Bivand and Wong, 2018). We used a two-sided significance test (α = 0.05).

### 2.3 Precipitation and temperature conditions

We used 5km × 5km grids of spatially kriged, monthly mean precipitation, maximum temperature, and minimum temperature spanning 2007–2020 from the Bureau of Meteorology (Australian Government Bureau of Meteorology, 2020). We converted these monthly grids to points and intersected the points with the 20 km roost foraging buffers. We then calculated a spatial mean of the precipitation, minimum temperature, and maximum temperature anomaly point values within the 20 km foraging areas of each roost each month. We also calculated a 3-month moving average for each variable. We then associated each roost survey with the 3-month moving average weather conditions experienced in that roost’s buffer over the prior 12 months.

### 2.4 Environmental interactions with mixed effects modeling

We used generalized logistic regression mixed-effects models to investigate the dynamic relationship between black flying fox presence at roosts in winter and the 1) weather conditions and 2) typical and atypical winter habitat. Mixed effects models are broadly applicable to ecological systems (Bolker, 2015) and allow for non-linear functions to be fit to data. In our case, we can assess if the fixed effects, such as the interactions of precipitation conditions and typical and atypical winter habitats, influence winter roost occupancy of black flying foxes while also including random effects. This allows us to account for the wide spatiotemporal variation in winter surveys at flying fox roosts. The presence and absence of black flying foxes (ρ) at observed roosts were fit with logistic regression models, with *i* indicating temporal surveys and roost name (*j*), *x*_1ij_ *x*_2ij_ indicating an interaction between environmental variables at roosts, and (ζ) included as random effects.


$$\text{logit}\left(\rho_{ij}\right)=\beta_{0}+x_{1ij}x_{2ij}+\zeta_{j}$$

We fit multivariate regression models with the *glmmLasso* package (v1.6.2) to perform multivariate predictor selection on all environmental variables described (Groll and Tutz, 2014). We used roost names as a random effect in the model, to account for inconsistent roost sampling over time. Fixed effects that had a correlation value greater than 0.6 with another factor in the final model were removed. We optimized lambda (λ), the shrinkage term used by the glmmLasso function to perform model variable selection. We tested each model with λ values ranging from 0 to 100,000 in 10 log steps for every model. We then ran the model with the optimized λ value and ran predictors identified as significant from the glmmLasso function into a glmer function in the *lme4* package (v1.1–33) (Bates et al., 2015). We examined the results for the simplest model with the lowest BIC from our multivariate model selection process. All spatial analyses were performed in the GDA 1994 Australian Albers projection in ArcGIS Pro 3.0 (ESRI, 2023). We performed statistical analyses, modeling, and data visualizations in R (R Core Team, 2019).

## 3 Results

**FIGURE 2:**
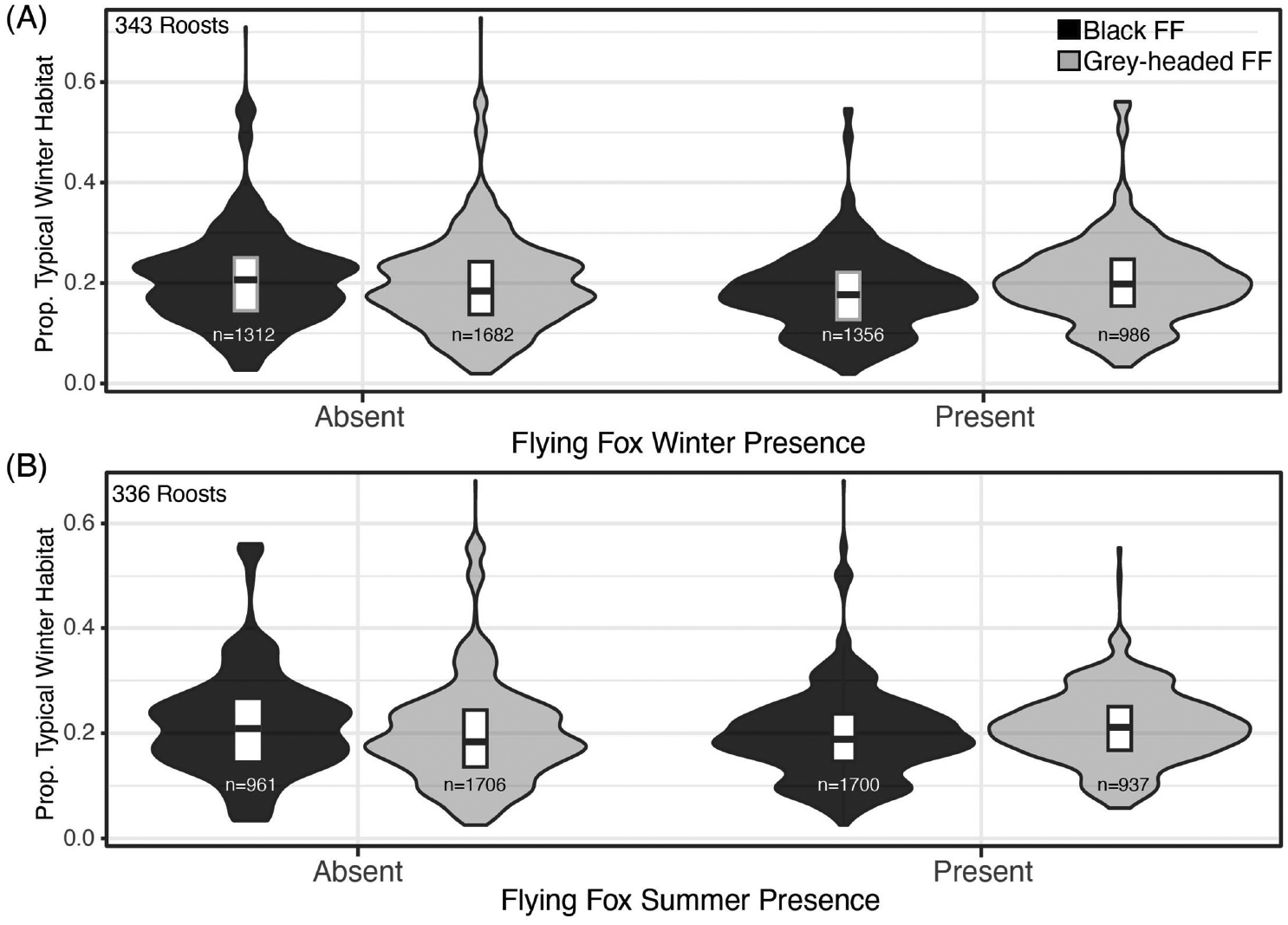
Proportion of Winter Habitats and Moran’s I Values by Black Flying Fox Roost Occupancy. (**A**) The proportion of winter habitat extent in roosts’ 20km foraging buffers, for roosts where black flying foxes and grey-headed flying were absent (left) and present (right) in winter months from 2007–2020. (**B**) Black and grey-headed flying fox absence and presence in summer months, 2007–2020.

**FIGURE 3:**
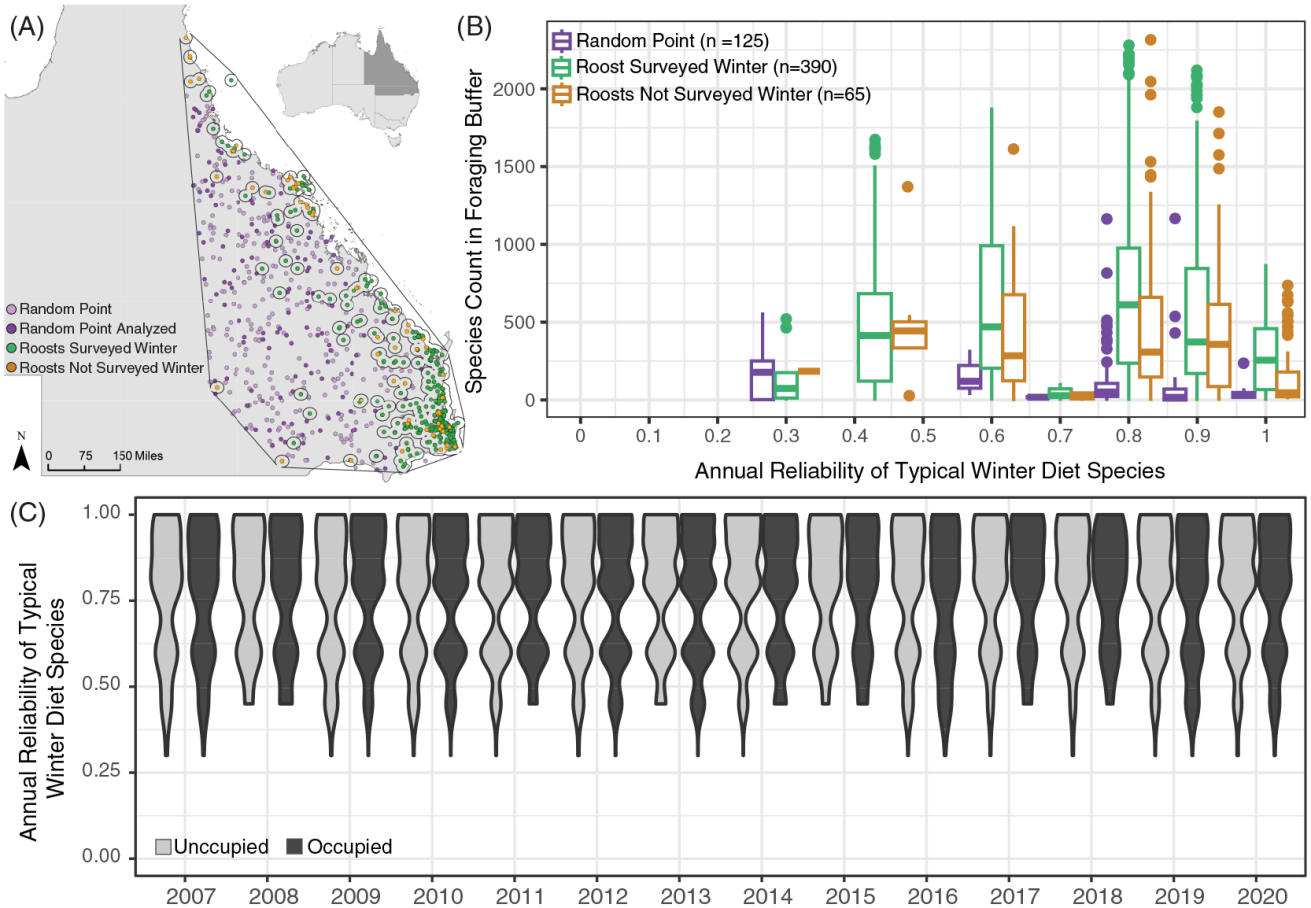
Annual reliability scores of the most frequent typical winter diet species in roost foraging areas, plotted by roost occupancy, and randomly generated points. (**A**) Map of Queensland identifying flying fox roosts surveyed in winter between 2007 and 2020 (green circles), roosts not surveyed in winter (orange circles), randomly generated points (light purple circles), and the randomly generated points analyzed (dark purple circles) with the hull of flying fox roosts outlined in black. Empty circles around roosts denote the 20km foraging area. (**B**) Frequency of fruiting or flowering for the six most prevalent winter diet species in a 20km radius buffer around flying fox roosts (green, orange) and randomly generated points (purple) in the geometric hull around all roosts. (**C**) Violin plots of the annual reliability score of typical winter diet species in roosts that were unoccupied (light grey) and occupied (dark grey) in the winter.

Black flying foxes occupied roosts with a wide range of typical winter habitat extents, and, to a lesser degree, atypical winter habitat extent, in their proximal foraging areas. Black flying fox winter roost occupancy was not explained by the amount of winter habitat in the 20km foraging area (Table 1, Figure 2A), the spatial arrangement of those winter habitats (Table 2; Supplementary Figure S4), or the reliability of those winter resources (Figure 3C). However, the frequency at which annually reliable winter diet species occur in foraging areas may influence where roosts are established on the landscape (Figure 3B). We also found no evidence that interactions between the average past weather conditions and winter habitat attributes explained winter roost occupancy (Supplementary Figure S5). Black flying fox winter roost presence was extremely dynamic and poorly correlated to static land metrics of vegetation presence, distribution, and reliability.

### 3.1 Winter habitat in roost foraging areas

All roosts contained typical winter habitats, however atypical winter resources were scarcer and were not documented near every roost (Table 1). Atypical winter habitat in roost foraging areas was largely known from observations from GBIF (i.e. single tree locations), rather than vegetation polygons from the VMRE maps. These data sources expectedly constrained the extent of atypical winter habitats we identified in roost foraging areas.

The proportion of typical and atypical winter foraging habitats did not influence winter roost occupancy of black flying foxes (Figure 2A). The average proportions of typical winter foraging habitats were similar at occupied and unoccupied roosts for both black and ecologically similar grey-headed flying fox in multiple seasons (Figures 2A, B). All flying fox roosts in Queensland were found to have dispersed winter habitats with atypical winter habitats being more dispersed than typical winter habitats in roost foraging areas (Table 2). We found the spatial arrangement of winter habitats, as measured by Moran’s I, was mostly correlated with the proportion of winter habitat in the foraging area (Supplementary Figure S4) and did not explain the winter presence of black flying foxes in surveyed roosts. Moran’s I was also largely determined by the foraging buffer size used (Supplementary Figure S3).

We found that the annual reliability of the most prevalent winter diet species around each roost did not explain the winter roost occupancy of black flying foxes (Figure 3C) but may explain where flying fox roosts are established (Figure 3B). We observed a clear relationship between the reliability of species in roost foraging areas and the locations of flying fox roosts on the landscape. The frequency of winter diet species with relatively high annual reliability scores was significantly higher in 20km areas around flying fox roosts in the NFFMP compared to random points generated on the landscape (p-val <0.005) (Figure 3C).

Roosts with similar species’ reliability scores of typical winter diet species were observed both occupied and unoccupied in various winters. Atypical winter resources included here are primarily fruit species, which produce fruits annually (House, 1992; Westcott et al., 2005; Hawkins et al., 2018; Business Queensland, 2021). This limited the variation in reliability scores for atypical resources for occupied and unoccupied roosts, and we found no explanatory power in the reliability of atypical winter resources for roost occupancy. We calculated greater variation in the range of typical winter reliability scores, but we saw no relationship between the reliability of species and black flying fox roost occupancy.

### 3.2 Environmental interactions and black flying fox winter occupancy

The final mixed-effects model investigating the environmental interactions identified four statistically significant relationships for winter roost occupancy between the Moran’s I value of winter habitats and past weather conditions at roosts (Table 3). The model identified significant interactions between the Moran’s I values for typical and atypical winter habitats and precipitation and temperatures from the previous 12 months as influencing black flying fox winter roost occupancy (Table 3). However, further investigation of these relationships in the data did not support the relationship identified in the model. We found that the relationship between winter roost occupancy and the Moran’s I value for both winter habitat types was similar across the gradient of all rainfall levels the 12 months prior (Supplementary Figure S5). These data conflict with our model results, suggesting these significant interactions were more likely a product of overfitting, rather than a true biological relationship in the environment.

## 4 Discussion

This study examined the relationships between winter habitat type, abundance, reliability, spatial distribution, and black flying fox occupancy at roosts in winter months, the season when Hendra virus spills over most frequently in eastern Queensland. We found that black flying fox winter roost presence was not impacted by the abundance of typical or atypical winter habitats in the 20 km foraging buffer (Figure 2A). This relationship was also consistent when we examined the grey-headed flying fox winter presence and the presence of both flying fox species in summer months (Figure 2B) as well as other foraging radii (Supplementary Figure S2). We found all roosts were located near winter habitat that was dispersed, as noted by negative Moran’s I values. We likewise found that all roosts were near patches that contained annually reliable typical winter diet species in foraging areas, and reliability scores similarly did not differ between roosts that were occupied or unoccupied. The statistically significant interactions between the weather conditions in the previous spring and winter and the various winter habitat attributes identified in the mixed-effects model were not supported by a close examination of those variable relationships (Supplementary Figure S5). Patterns of black flying fox winter occupancy were similar across all ranges of precipitation conditions. We found that the winter presence of black flying foxes was interannually dynamic, and the spatial land metrics quantified here could not explain those dynamics.

Our analyses were unable to explain much of black flying fox winter roost presence and absence, likely due to complex drivers influencing their winter distribution. As dietary generalists (Palmer, 1997), it’s possible black flying foxes do not need to find optimal foraging habitats, and instead roost in areas that are merely sufficient enough. A study of black flying foxes from 2012–2017 found weak support that foraging movements follow seasonal environmental cues (Welbergen et al., 2020); black flying fox movement may no longer be as strongly influenced by seasonal foraging resources as it once historically was. Our work did not support the suggestion that flying foxes may be occupying roosts with access to more atypical (i.e. non-native) diet resources due to their increased spatiotemporal availability or productivity (Figure 3C). Our results suggest the abundance of winter habitat is not as relevant for flying foxes as the frequency of winter diet species in foraging areas (Figure 3B). Roosts with high frequencies of diet species may increase the chance for individuals to encounter irregular and geographically focused bursts of productivity in winter habitats, which is likely the strongest driver of flying fox winter roosting presence (Giles et al., 2016). Unfortunately, estimating winter flowering for eucalypts across this scale is challenging, and accounting for all the potential resources in anthropogenically modified environments, such as fruit trees in residential yards, is not possible.

It’s likely that our data sources and methodology overestimated winter habitat extent and biased the relationship evaluated here. We defined winter habitat as the presence of diet species in a vegetation community, but we did not have information on the relative abundance or spatial distribution of the diet species within a patch. We likely defined some areas as ‘typical winter habitat’ that may not have been sufficiently abundant or productive to attract foraging black flying foxes in winter. Additionally, the abundance and distribution of atypical winter habitats were drastically constrained by the availability of geospatial data describing their locations. Atypical winter diet species located far from human settlements or in areas not commonly visited by humans are unlikely to be captured in the GBIF database. Varying levels of roost survey intensity in the NFFMP (Supplementary Figure S1) may have contributed to the weak relationship between the environment and roost presence identified here. Finally, the temporal gaps around the biennial vegetation data used here may not reflect actual winter habitat abundance at roost locations during the time of the survey if conditions were changing rapidly. Collectively, these limitations could have contributed to our findings of weak environmental factors associated with winter roost occupancy. More examination of black flying fox foraging movement will help characterize the predominant vegetation types used for foraging resources in winter. This can inform management efforts on strategies to minimize winter food shortages for flying foxes, which may reduce the risk of Hendra virus spillover.

Ecological solutions for spillover prevention, such as conservation of existing habitats and replanting of winter diet resources, have also been proposed as a way to increase resource abundance and bat foraging activity in native forests instead of agricultural and anthropogenic lands (Giles et al., 2018; Eby et al., 2023). This solution would also aid forest health and benefit other wildlife species such as birds and small marsupials (Eby, 2016). However, our findings do not indicate that the extent or spatial distribution of winter foraging habitats strongly influences winter black flying fox presence. Further, some flying fox diet species take 5–10 years to become mature and produce flowers so these solutions would not resolve immediate problems (Law et al., 2000). More rapid solutions in addition to vegetation management should be considered to support flying foxes during winter food shortages.

Supplementing the availability of fruits or high-quality, native nectar for flying fox populations may be a more immediate way to ensure there is sufficient food in winter and mitigate the potential for a food shortage. While supplemental feeding programs for wildlife may lead to unintended consequences such as aggression or disease transmission (Forristal et al., 2012; Sorensen et al., 2014; Jones et al., 2014), Hendra virus infection in bats was not found to be related to roost size or density (Lunn et al., 2022). The aggregation of flying foxes at feeding sites would likely not necessarily increase viral transmission in the population. Feeding locations could also be strategically focused away from urban and peri-urban areas to help spatially separate bats from human systems and minimize the proximity to agricultural lands with horses. Supplemental feeding done carefully could mimic the conditions of large productive blooms of native eucalypt trees, which would not increase Hendra virus risk. Additional interventions that limit horse exposure to bat excreta and horse vaccinations would have direct impacts further minimizing the risk of Hendra spillover to horses.

The need for a thorough investigation of environmental conditions that are important for reservoir hosts broadly applies to many emerging viruses, including Nipah virus and Ebola virus. These bat-borne viruses have caused outbreaks in humans with case fatality rates as high as 90%. Both of these viruses have been linked to the conversion of forested bat habitats to anthropogenically managed lands (Hsu et al., 2004; Gurley et al., 2017; Rulli et al., 2017; Olivero et al., 2017). To reduce bat-borne viral spillovers, it will be imperative to identify the factors that drive bat movements. Whether human-driven changes negatively impact the reservoir hosts’ health, increase the frequency of contact between humans and wildlife, or both, disentangling the influences of these processes will help explain how and why zoonotic pathogens emerge.

## Supplementary Material

Supplementary Methods

## Figures and Tables

**TABLE 1:** Proportions of typical and atypical winter habitat extents in roosts’ 20 km foraging buffers surveyed by the NFFMP in 2007.

In 20km Foraging Buffers, 2007	Average	Median	Range
Proportion Typical Winter Habitat	0.2093	0.1870	0.0192 – 0.7277
Proportion Atypical Winter Habitat	0.0236	0.0048	0 – 0.2448

**TABLE 2:** R-squared values of the linear relationships between the proportion of typical and atypical winter habitat in 20 km roosts foraging buffers and the Moran’s I value of roosts’ winter habitats for roosts that were surveyed by the NFFMP each year from 2007–2020, grouped by black flying fox occupancy status.

Roost Observations (n= 2,668)	BFF Occupancy	Average R^2^ of Linear Models (Prop. Typical Winter Habitats vs. Moran’s I)	Range of R^2^ of Linear Models (Prop. Typical Winter Habitats vs. Moran’s I)	Average R^2^ of Linear Models (Prop. Atypical Winter Habitats vs. Moran’s I)	Range of R^2^ of Linear Models (Prop. Atypical Winter Habitats vs. Moran’s I)
Winters only from 2007–2020	Absent	0.3262	0.0349 – 0.5027	0.5453	0.4905 – 0.6823
Winters only from 2007–2020	Present	0.2784	0.0355 – 0.5456	0.4987	0.3249– 0.7685

**TABLE 3:** Multivariate logistic mixed effects regression significant results.

Predictor	Estimate	St.error	Zvalue	P-value	Significance
Moran’s I Typical Winter * Mean Precipitation (3mon. average 12 months before survey)	54.419	21.616	2.518	0.01182	*
Moran’s I Typical Winter * Mean Max Temp (3mon. average 12 months before survey)	−49.833	20.887	−2.386	0.01704	*
Moran’s I Atypical Winter * Mean Precipitation (3mon. average 12 months before survey)	−32.978	12.452	−2.648	0.00809	**
Moran’s I Atypical Winter: * Mean Max Temp (3mon. average 12 months before survey)	28.756	11.525	2.495	0.01259	*

Estimates and p-values for multivariate logistic mixed effects model for winter roost occupancy on the environmental interactions between the Moran’s I for typical and atypical winter habitats and current or preceding weather conditions, (*) indicates a p-value <0.05.

Only significant predictors from model are shown.

## Data Availability

The datasets presented in this study can be found in online repositories. The names of the repository/repositories and accession number(s) can be found below: https://github.com/bhartilab/BFFseasonal_occ_20072020.
