# Supplementary material for: Native and non-native winter foraging resources do not explain Pteropus alecto winter roost occupancy in Queensland, Australia
Source: Front Ecol Evol. Author manuscript; Available in PMC 2024 Dec 18. (PMC11654838; doi:10.3389/fevo.2024.1483865)
Supplement: Supplementary Methods [file NIHMS2041653-supplement-Supplementary_Methods.docx]

**Supplementary Methods**

*Black Flying Fox Winter Habitat Selection*

To identify vegetation communities that contained black flying fox winter diet resources, we used the Regional Ecosystem Description Database (REDD) Version 12 from the Queensland Herbarium (Queensland Herbarium 2019). Processing of this database is described in the supplementary methods of Baranowski and Bharti 2023. Typical winter diet species were derived from early descriptions of flying fox diets from Ratcliffe 1931. Ratcliffe states the main diet of flying foxes is “undoubtedly blossom” of (*Eucalyptus, Angophora, Syncarpia,* etc.) and describes how rainforest fruits (*Ficus*) and cultivated fruit (Citrus, Prunus) are foraged on to a lesser degree. Attacks on pome, stone, and citrus fruits were observed when there were “temporary disturbances to the local supply of ‘natural’ food” (Ratcliffe 1931). We created our lists of typical and atypical winter diet species based on the genera and species observed to be foraged on by flying foxes in winter by Ratcliffe 1931.

We identified regional ecosystems that contained at least one typical or atypical winter diet species, as observed from studies on black flying fox diet (Ratcliffe 1931; Markus and Hall 2004; Eby and Law 2008; Field et al. 2016; Eby et al. 2019; Griffith 2020; Bell et al. 2021; Bradford et al. 2022). See Table S1 for a complete list of typical and atypical black flying fox winter diet species. Since the abundance of productive vegetation within a patch is important for flying foxes (Eby and Law 2008), we only included regional ecosystems where the diet species of interest was listed within the top five species by relative abundance. This was to identify areas where these typical species were relatively ‘dominant’ or potentially of reasonable abundance to be useful for black flying fox populations.

We identified single-point locations of typical and atypical diet species using the Global Biodiversity Information Facility (Derived dataset GBIF.org 2024). When exact species names of diet species were not provided in the literature (e.g. persimmons, apples), we searched the genus of the diet species in GBIF (e.g. *Diospyros, Malus*) and included all species with records in Queensland, Australia. We then limited records by spatial resolution of the location, removing data with less than 2 points of decimal degrees in their latitude and longitude, and aggregated records to remove duplicate identifications of the same tree. We removed trees recorded before the year 2000 that were outside the spatial extent of the National Woody Vegetation Dataset in 2000 (Department of Environment and Science 2020), to account for the likely deforestation of these records before our study period.

*List of Black Flying Fox Winter Diet Species*

| Genus | Species | Source of Foraging Evidence | Diet Type | Annual Reliability | Source for reliability |
| --- | --- | --- | --- | --- | --- |
| *Acmena* | *hemilampra* | Eby & Law 2008 | atypical |  |  |
| *A.* | *ingens* | Eby & Law 2008 | atypical |  |  |
| *A.* | *smithii* | Eby & Law 2008 | atypical |  |  |
| *Alphitonia* | *excelsa* | Eby & Law 2008, Griffith 2020 | atypical |  |  |
| *Archontophoenix* | *cunninghamiana* | Eby & Law 2008, Griffith 2020 | atypical |  |  |
| *Asparagus* | *africanus* | Markus & Hall 2004, Field et al. 2016 | atypical | 1 | Living Atlas of Australia |
| *Avicennia* | *marina* | Eby & Law 2008, Griffith 2020 | atypical |  |  |
| *Callistemon* | *viminalis* | Eby, Sims, & Bracks 2019, Bradford et al. 2022 personal comm. | atypical |  |  |
| *Cissus* | *hypoglauca* | Eby & Law 2008, Griffith 2020 | atypical |  |  |
| *Citrus* | *aurantium* | Ratcliffe 1931, Griffith 2020 | atypical |  |  |
| *C.* | *limon* | Ratcliffe 1931, Griffith 2020 | atypical |  |  |
| *C.* | *reticulata* | Ratcliffe 1931, Griffith 2020 | atypical |  |  |
| *C.* | *sinensis* | Ratcliffe 1931, Griffith 2020 | atypical |  |  |
| *Dendrocnide* | *excelsa* | Eby & Law 2008, Griffith 2020 | atypical |  |  |
| *Diospyros* | *australis* | Ratcliffe 1931 | atypical | 1 | House 1992 |
| *D.* | *calycantha* | Ratcliffe 1931 | atypical | 1 | House 1992 |
| *D.* | *compacta* | Ratcliffe 1931 | atypical | 1 | House 1992 |
| *D.* | *fasciculosa* | Ratcliffe 1931 | atypical | 1 | House 1992 |
| *D.* | *geminata* | Ratcliffe 1931 | atypical | 1 | House 1992 |
| *D.* | *granitica* | Ratcliffe 1931 | atypical | 1 | House 1992 |
| *D.* | *hebecarpa* | Ratcliffe 1931 | atypical | 1 | House 1992 |
| *D.* | *hemicycloides* | Ratcliffe 1931 | atypical | 1 | House 1992 |
| *D.* | *humilis* | Ratcliffe 1931 | atypical | 1 | House 1992 |
| *D.* | *kaki* | Ratcliffe 1931 | atypical | 1 | House 1992 |
| *D.* | *laurina* | Ratcliffe 1931 | atypical | 1 | House 1992 |
| *D.* | *littorea* | Ratcliffe 1931 | atypical | 1 | House 1992 |
| *D.* | *mabacea* | Ratcliffe 1931 | atypical | 1 | House 1992 |
| *D.* | *maritima* | Ratcliffe 1931 | atypical | 1 | House 1992 |
| *D.* | *peninsularis* | Ratcliffe 1931 | atypical | 1 | House 1992 |
| *D.* | *pentamera* | Ratcliffe 1931 | atypical | 1 | House 1992 |
| *D.* | *pluviatilis* | Ratcliffe 1931 | atypical | 1 | House 1992 |
| *D.* | *uvida* | Ratcliffe 1931 | atypical | 1 | House 1992 |
| *D.* | *yandina* | Ratcliffe 1931 | atypical | 1 | House 1992 |
| *Diploglottis* | *australis* | Eby & Law 2008 | atypical |  |  |
| *Ehretia* | *acuminata* | Eby & Law 2008 | atypical |  |  |
| *Elaeocarpus* | *grandis* | Eby & Law 2008 | atypical | 1 | Westcott et al. 2005 |
| *E.* | *obovatus* | Eby & Law 2008 | atypical |  |  |
| *E.* | *reticulatus* | Eby & Law 2008 | atypical |  |  |
| *Eriobotrya* | *japonica* | Markus & Hall 2004 | atypical |  |  |
| *Hedycarya* | *angustifolia* | Eby & Law 2008 | atypical |  |  |
| *Ligustrum* | *lucidum* | Griffith 2020 | atypical |  |  |
| *Livistona* | *australis* | Eby & Law 2008, Griffith 2020 | atypical |  |  |
| *Maclura* | *cochinchinensis* | Eby & Law 2008 | atypical |  |  |
| *Mallotus* | *discolor* | Eby & Law 2008 | atypical |  |  |
| *Malus* | *domestica* | Ratcliffe 1931 | atypical | 1 | Ratcliffe 1931 |
| *M.* | *pumila* | Ratcliffe 1931 | atypical | 1 | Ratcliffe 1931 |
| *Mangifera* | *indica* | Ratcliffe 1931, Markus &. Hall 2004, Bradford personal comm., from Bell et al. 2021& Bradford et al. 2022 | atypical | 1 | Ratcliffe 1931 |
| *Melia* | *azedarach* | Bradford personal comm., from Bell et al. 2021& Bradford et al. 2022, Eby & Law 2008, Griffith 2020 | atypical |  |  |
| *Melodinus* | *australis* | Eby & Law 2008 | atypical |  |  |
| *Morinda* | *jasminoides* | Eby & Law 2008 | atypical |  |  |
| *Notothixos* | *cornifolius* | Eby & Law 2008 | atypical |  |  |
| *Passiflora* | *herbertiana* | Eby & Law 2008 | atypical |  |  |
| *Pennantia* | *cunninghamii* | Eby & Law 2008 | atypical |  |  |
| *Pittosporum* | *undulatum* | Eby & Law 2008 | atypical |  |  |
| *Planchonella* | *australis* | Eby & Law 2008 | atypical |  |  |
| *Podocarpus* | *elatus* | Eby & Law 2008, Griffith 2020 | atypical |  |  |
| *Polyosma* | *cunninghamii* | Eby & Law 2008 | atypical |  |  |
| *Prunus* | *armeniaca* | Ratcliffe 1931 | atypical | 1 | Ratcliffe 1931 |
| *P.* | *avium* | Ratcliffe 1931 | atypical | 1 | Ratcliffe 1931 |
| *P.* | *cerasifera* | Ratcliffe 1931 | atypical | 1 | Ratcliffe 1931 |
| *P.* | *domestica* | Ratcliffe 1931 | atypical | 1 | Ratcliffe 1931 |
| *P.* | *persica* | Ratcliffe 1931 | atypical | 1 | Ratcliffe 1931 |
| *P.* | *rivularis* | Ratcliffe 1931 | atypical | 1 | Ratcliffe 1931 |
| *P.* | *cattleyanum* | Ratcliffe 1931 | atypical | 1 | Ratcliffe 1931 |
| *Psidium* | *guajava* | Griffith 2020 | atypical | 1 | Ratcliffe 1931 |
| *Rauwenhoffia* | *leichardtii* | Eby & Law 2008 | atypical |  |  |
| *Rhaphiolepis* | *loquata* | Eby & Law 2008 | atypical |  |  |
| *Rhodamnia* | *argentea* | Eby & Law 2008 | atypical |  |  |
| *Rubus* | *rosifolius* | Eby & Law 2008 | atypical |  |  |
| *Sambucus* | *australasica* | Eby & Law 2008 | atypical |  |  |
| *Schefflera* | *actinophylla* | Markus & Hall 2004 | atypical |  |  |
| *Schizomeria* | *ovata* | Eby & Law 2008 | atypical |  |  |
| *Solanum* | *aviculare* | Eby & Law 2008 | atypical |  |  |
| *S.* | *mauritianum* | Field et al. 2016, Griffith 2020, Griffith 2020 | atypical |  |  |
| *S.* | *seaforthianum* | Field et al. 2016 | atypical | 1 | Ratcliffe 1931 |
| *Syagrus* | *romanzoffiana* | Markus & Hall 2004, Field et al. 2016 | atypical | 1 | Ratcliffe 1931 |
| *Syzygium* | *australe* | Eby & Law 2008 | atypical | 1 | Ratcliffe 1931 |
| *S.* | *corynanthum* | Eby & Law 2008 | atypical | 1 | Ratcliffe 1931 |
| *S.* | *crebrinerve* | Eby & Law 2008 | atypical | 1 | Ratcliffe 1931 |
| *S.* | *oleosum* | Eby & Law 2008 | atypical | 1 | Ratcliffe 1931 |
| *Ziziphus* | *mauritiana* | Bradford personal comm., from Bell et al. 2021 & Bradford et al. 2022 | atypical | 1 | Ratcliffe 1931 |
| *Banksia* | *aemula* | Ratcliffe 1931, Eby, Sims, & Bracks 2019 | typical |  |  |
| *B.* | *integrifolia* | Ratcliffe 1932, McWilliams 1986, Eby & Law 2008, Eby, Sims, & Bracks 2019 | typical | 1 | Eby & Law 2008 |
| *Coymbia* | *citriodora* | Ratcliffe 1931, Ratcliffe 1932, Eby & Law 2008 | typical | 0.3 | Eby & Law 2008 |
| *C.* | *maculata* | Ratcliffe 1931, Eby & Law 2008, Eby, Sims, & Bracks 2019, McWilliams 1986 | typical |  |  |
| *Eucalyptus* | *albens* | Eby & Law 2008, Eby, Sims, & Bracks 2019 | typical | 0.7 | Eby & Law 2008 |
| *E.* | *camaldulensis* | Eby & Law 2008, Eby, Sims, & Bracks 2019, Bradford personal comm., from Bell et al. 2021 & Bradford et al. 2022 | typical |  |  |
| *E.* | *conica* | Eby & Law 2008, Eby, Sims, & Bracks 2019 | typical |  |  |
| *E.* | *crebra* | Bradford personal comm., from Bell et al. 2021 & Bradford et al. 2022 | typical |  |  |
| *E.* | *fibrosa* | Eby & Law 2008 | typical | 0.3 | Eby & Law 2008 |
| *E.* | *fusiformis* | Eby, Sims, & Bracks 2019 | typical |  |  |
| *E.* | *melliodora* | Eby & Law 2008 | typical | 0.3 | Eby & Law 2008 |
| *E.* | *ochrophloia* | Eby, Sims, & Bracks 2019 | typical |  |  |
| *E.* | *pilularis* | Eby & Law 2008 | typical | 0.45 | Eby & Law 2008 |
| *E.* | *platyphylla* | Bradford personal comm., from Bell et al. 2021 & Bradford et al. 2022 | typical |  |  |
| *E.* | *robusta* | Eby & Law 2008, Eby, Sims, & Bracks 2019 | typical | 1 | Eby & Law 2008 |
| *E.* | *seeana* | Eby & Law 2008, Eby, Sims, & Bracks 2019 | typical | 0.8 | Eby & Law 2008 |
| *E.* | *siderophloia* | Eby & Law 2008, Eby, Sims, & Bracks 2019 | typical | 0.6 | Eby & Law 2008 |
| *E.* | *sideroxlyon* | Eby & Law 2008, Eby, Sims, & Bracks 2019 | typical | 0.3 | Eby & Law 2008 |
| *E.* | *tereticornis* | Ratcliffe 1931, Ratcliffe 1932, Eby & Law 2008, Eby, Sims, & Bracks 2019, Bradford et al. 2022 | typical | 0.8 | Eby & Law 2008 |
| *E.* | *tetrapleura* | Eby, Sims, & Bracks 2019 | typical |  |  |
| *Ficus* | *benjamina* | Markus & Hall 2004, Bradford personal comm., from Bell et al. 2021 & Bradford et al. 2022 | typical | 1 | Schmelitschek et al. 2009 |
| *F.* | *coronata* | Ratcliffe 1931, Eby & Law 2008 | typical | 1 | Schmelitschek et al. 2009 |
| *F.* | *fraseri* | Ratcliffe 1931, Eby & Law 2008 | typical | 1 | Schmelitschek et al. 2009 |
| *F.* | *macrophylla* | Ratcliffe 1931, Eby & Law 2008 | typical | 1 | Schmelitschek et al. 2009 |
| *F.* | *obliqua* | Eby & Law 2008, Markus & Hall 2004, Field et al. 2016 | typical | 1 | Schmelitschek et al. 2009 |
| *F.* | *racemosa* | Ratcliffe 1931 | typical | 1 | Schmelitschek et al. 2009 |
| *F.* | *rubiginosa* | Ratcliffe 1931, Eby & Law 2008 | typical | 1 | Schmelitschek et al. 2009 |
| *F.* | *superba* | Ratcliffe 1931, Eby & Law 2008 | typical | 1 | Schmelitschek et al. 2009 |
| *F.* | *virens* | Eby & Law 2008, Markus & Hall 2004 | typical | 1 | Schmelitschek et al. 2009 |
| *F.* | *watkinsiana* | Ratcliffe 1931, Eby & Law 2008 | typical | 1 | Schmelitschek et al. 2009 |
| *Melaleuca* | *fluviatilis* | Bradford personal comm., from Bell et al. 2021& Bradford et al. 2022, Griffith 2020 | typical |  |  |
| *M.* | *leucadendra* | Bradford personal comm., from Bell et al. 2021& Bradford et al. 2022, Griffith 2020 | typical |  |  |
| *M.* | *nervosa* | Bradford personal comm., from Bell et al. 2021& Bradford et al. 2022, Griffith 2020 | typical |  |  |
| *M.* | *quinquenervia* | Eby & Law 2008, Eby, Sims, & Bracks 2019, Griffith 2020 | typical | 1 | Eby & Law 2008 |
| *M.* | *viridiflora* | Bradford personal comm., from Bell et al. 2021, & Bradford et al. 2022 Palmer 1997 | typical | 0.8 | Eby & Law 2008 |

Table S1. List of typical and atypical winter diet species for black flying foxes.

**Supplementary Results**

*National Flying Fox Monitoring Surveys*

*
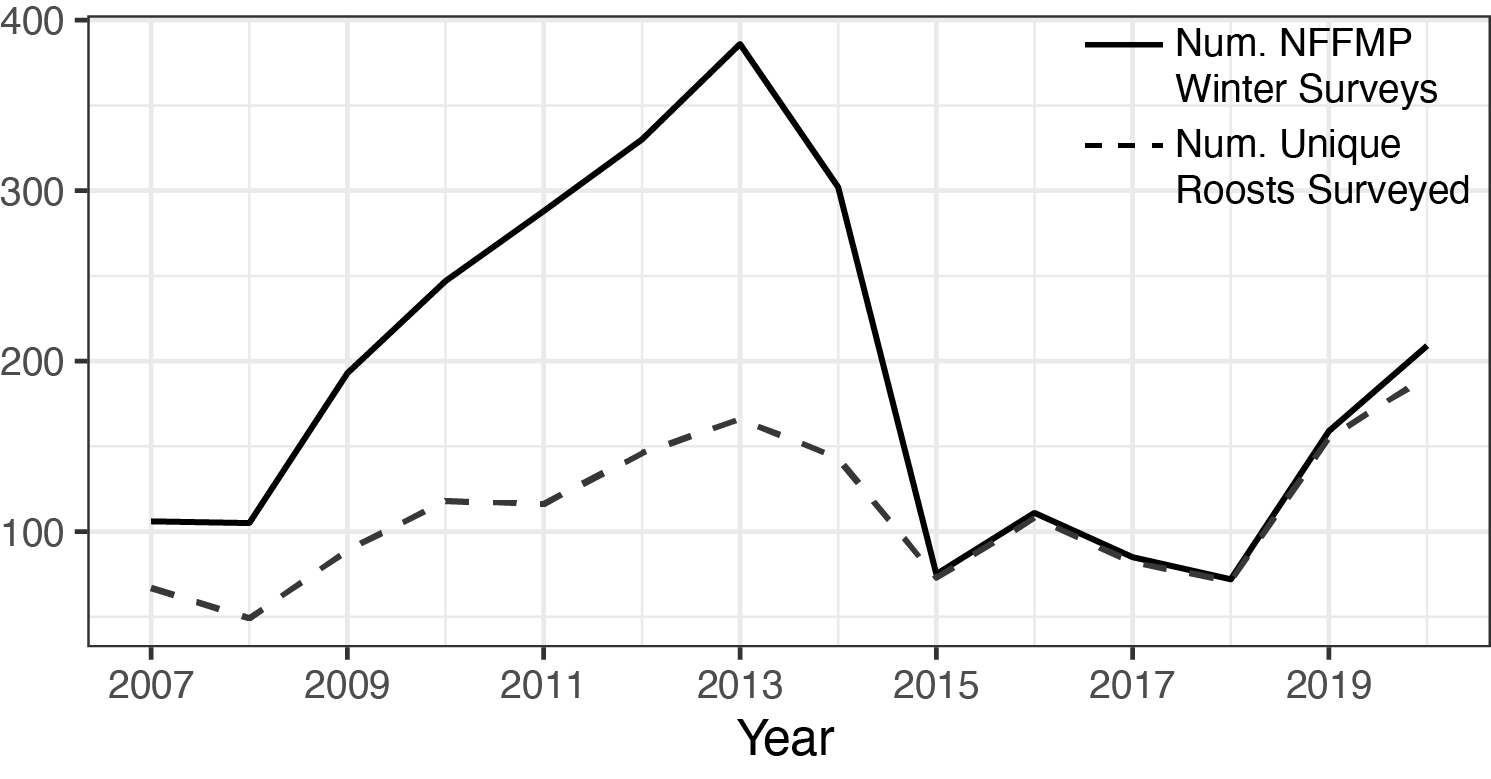
*

Figure S1. Number of surveys and unique roosts surveyed in the National Flying Fox Monitoring Program in Austral winter months (June, July, August) 2007-2020.

*Sensitivity Analysis on Buffer Size*

We performed a sensitivity analysis on the foraging buffer size to examine how spatial scale impacted the proportion of typical foraging winter habitat and Moran’s I value. We buffered all roosts by 10 km and 40 km radii and calculated the proportion of winter habitat and Moran’s I value of typical winter habitat in 2007 across these foraging areas.


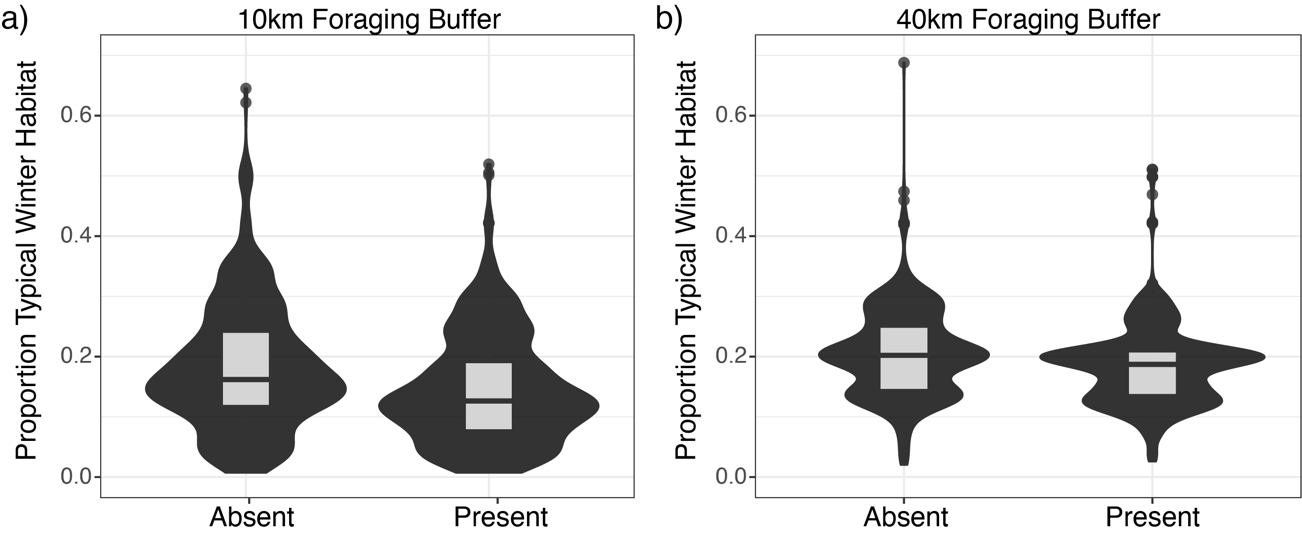


Figure S2. Proportion of Winter Habitats and Moran’s I Values by Black Flying Fox Roost Occupancy. The proportion of winter habitat extent in roosts’ 10 km (a) and 40 km (b) foraging buffers, for roosts where black flying foxes were absent (left) and present (right) in winter months from 2007-2020.

*
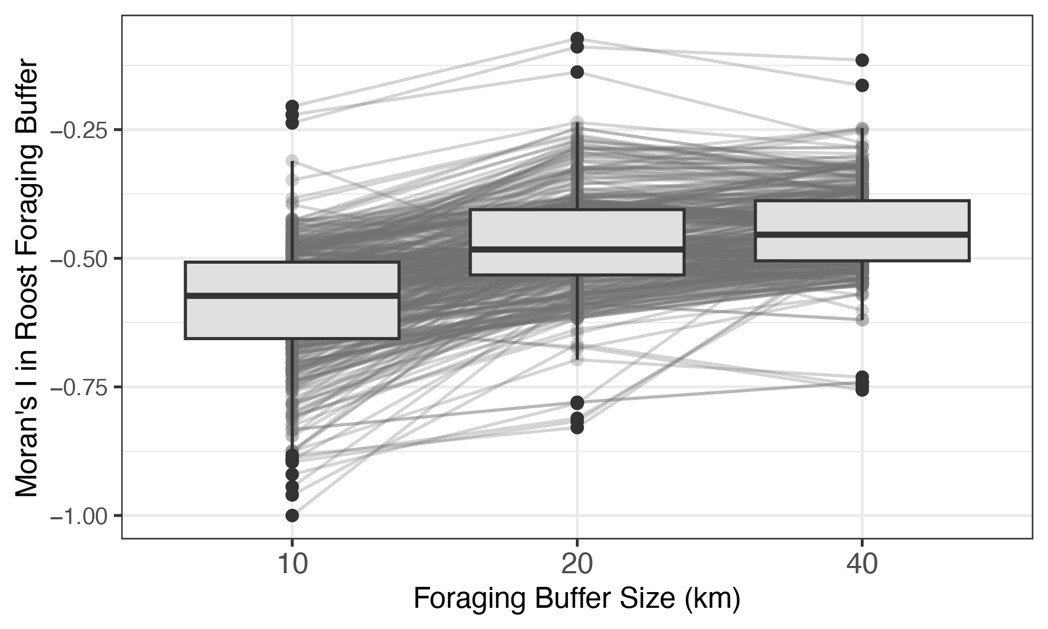
*

Figure S3. Moran’s I values for typical winter habitat in 2007 across foraging buffer radii of 10, 20 and 40 km around roosts. Values for the same roost are connected with a grey line.


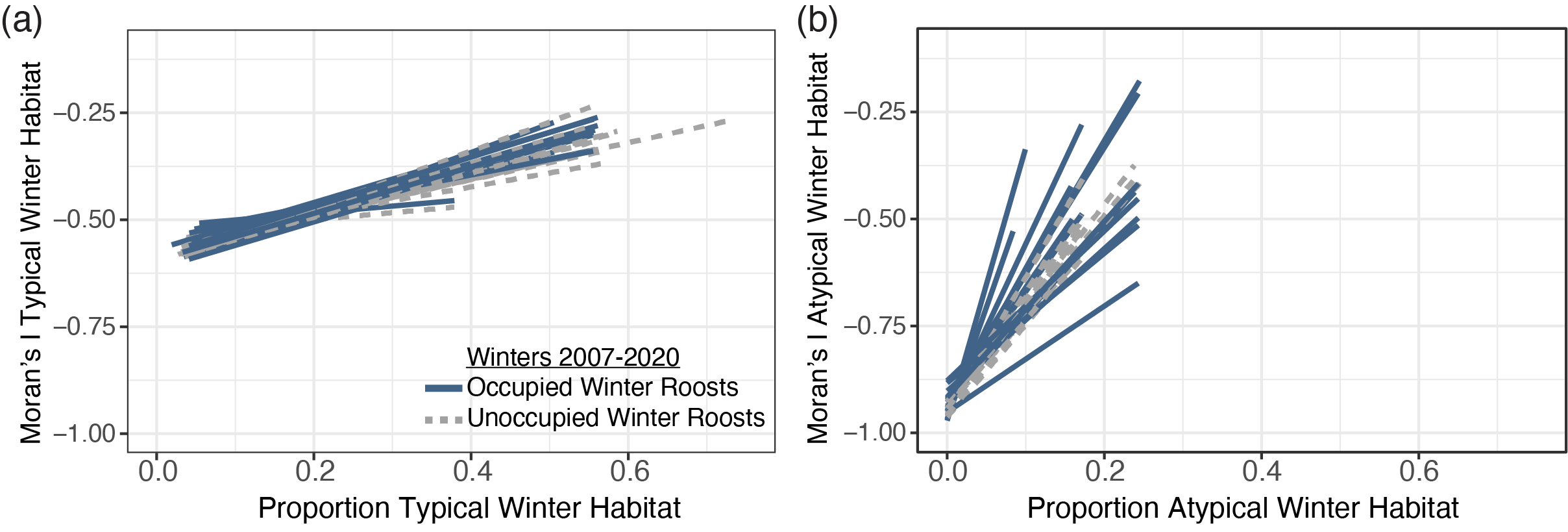


Figure S4. The Linear Relationships between Moran’s I and the Proportion of Winter Habitats. a) Relationship between Moran’s I of typical winter habitat and the proportion of typical winter habitat in roosts’ 20 km foraging buffers each year 2007-2020, colored by black flying fox occupancy. b) Relationship between Moran’s I for atypical winter habitat and the proportion of atypical winter habitat in roosts’ 20 km foraging buffers each year 2007-2020, colored by black flying fox occupancy.

We found that regardless of buffer size, typical winter habitat for black flying foxes is dispersed. Shrinking the foraging buffer size to radius 10 km resulted in the Moran’s I value for most roosts to decrease (Figure S3). Expanding the foraging buffer size to radius 40 km increased or decreased Moran’s I values depending on the roost. Although Moran’s I values for winter habitats did not explain black flying fox winter roost occupancy, these results highlight the importance of spatial scale when evaluating environmental drivers of animal presence.

*GLMM Model results*


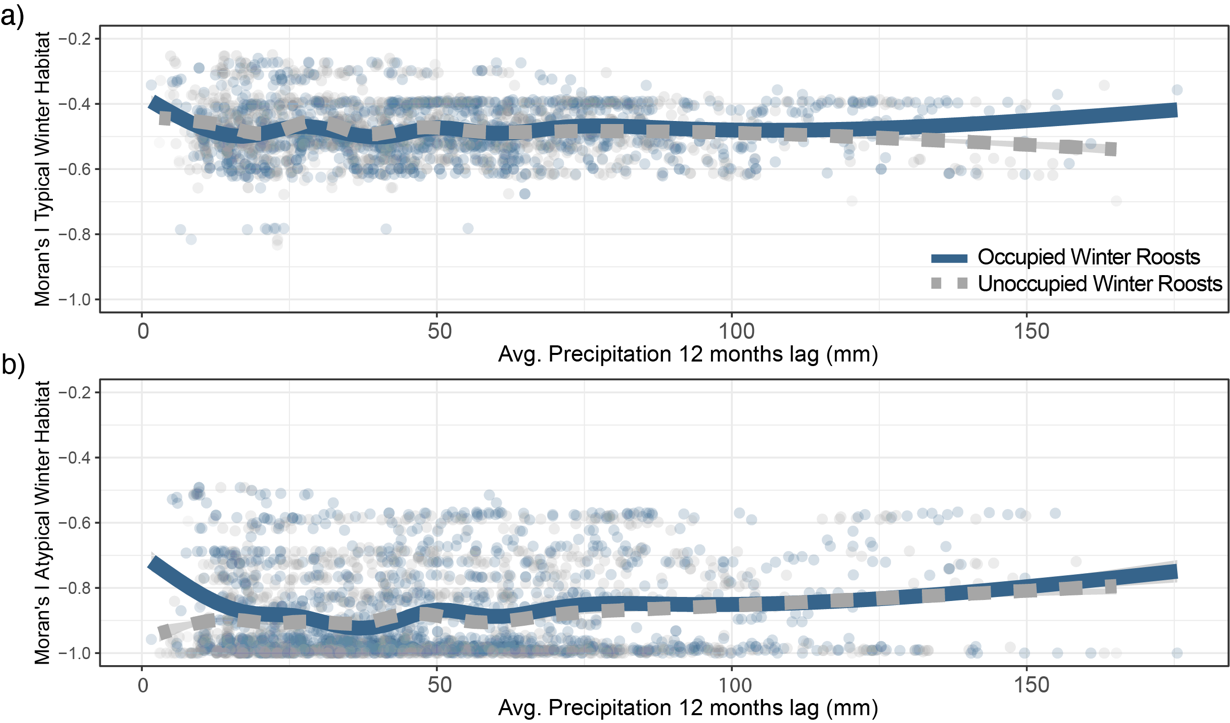


Figure S5. Relationship between Moran’s I of winter habitats and average precipitation conditions from 12 months prior. a) Generalized additive model (GAM) modeling of Black flying fox winter roost occupancy and Moran’s I of typical winter habitat at roosts by the average precipitation 12 months ago, colored by occupancy. b) Generalized additive model (GAM) model of winter occupancy and Moran’s I of atypical winter habitat at roosts by the average precipitation 12 months ago, colored by occupancy.

**References**

Baranowski K, Bharti N (2023) Habitat loss for black flying foxes and implications for Hendra virus. Landsc Ecol 1–14. https://doi.org/10.1007/s10980-023-01642-w

Bell KL, Batchelor KL, Bradford M, et al (2021) Optimisation of a pollen DNA metabarcoding method for diet analysis of fl ying-foxes ( Pteropus spp .). Aust J Zool

Bradford M, Venz M, Bell KL, et al (2022) The diet of a specialist nectarivore in Australia: The little red flying-fox (Pteropus scapulatus, Pteropodidae). Austral Ecol 47:619–628. https://doi.org/10.1111/aec.13143

Department of Environment and Science (2020) National Forest and Sparse Woody Vegetation Data (Version 5, 2020 Release). https://data.gov.au/data/dataset/national-forest-and-sparse-woody-vegetation-data-version-5-2020-release

Derived dataset GBIF.org (2024) Filtered export of GBIF occurrence data. https://doi.org/10.15468/dd.35ut8w

Eby P, Law B (2008) Ranking the feeding habitats of Grey-headed flying foxes for conservation management. A report for The Department of Environment and Climate Change (NSW) & The Department of Environment, Water, Heritage and the Arts

Eby P, Sims R, Bracks J (2019) Flying-fox Foraging Habitat Mapping NSW spatial patterns of habitat quality for flying-foxes

Field HE, Smith CS, De Jong CE, et al (2016) Landscape utilisation, animal behaviour and hendra virus risk. Ecohealth 13:26–38. https://doi.org/10.1007/s10393-015-1066-8

Griffith P (2020) Diet Partitioning in newly sympatric urban flying-foxes (Pteropus poliocephalus and Pteropus alecto). Aust Mammal 42:361–366

House SM (1992) Population Density and Fruit Set in Three Dioecious Tree Species in Australian Tropical Rain Forest. Source: Journal of Ecology 80:57–69

Living Atlas of Australia Asparagus africanus Lam. https://bie.ala.org.au/species/https://id.biodiversity.org.au/node/apni/2908730. Accessed 4 Aug 2024

Markus N, Hall L (2004) Foraging behaviour of the black flying-fox (Pteropus alecto) in the urban landscape of Brisbane, Queensland. Wildlife Research 31:345–355. https://doi.org/10.1071/WR01117

Queensland Herbarium (2019) Regional Ecosystem Description Database (REDD). Version 12.1. In: Queensland Department of Environment and Science: Brisbane. https://www.qld.gov.au/environment/plants-animals/plants/ecosystems/descriptions

Ratcliffe FN (1931) The Flying Fox (Pteropus) in Australia. Council for Scientific and Industrial Research

Schmelitschek E, French K, Parry-Jones K (2009) Fruit availability and utilisation by grey-headed flying foxes (Pteropodidae: Pteropus poliocephalus) in a human-modified environment on the south coast of New South Wales, Australia. Wildlife Research 36:592–600. https://doi.org/10.1071/WR08169

Westcott DA, Bradford MG, Dennis AJ, Lipsett-Moore G (2005) Keystone Fruit Resources and Australia’s Tropical Forests. In: Tropical Fruits and Frugivores: The Search for Strong Interactors. Springer, pp 237–260
